# Low Mismatch Rate between Double-Stranded RNA and Target mRNA Does Not Affect RNA Interference Efficiency in Colorado Potato Beetle

**DOI:** 10.3390/insects11070449

**Published:** 2020-07-16

**Authors:** Wanwan He, Wenbo Xu, Kaiyun Fu, Wenchao Guo, Jiang Zhang

**Affiliations:** 1 State Key Laboratory of Biocatalysis of Enzyme Engineering, School of Life Sciences, Hubei University, Wuhan 430062, China; hewanwan@stu.hubu.edu.cn (W.H.); wenboxu@stu.hubu.edu.cn (W.X.); 2 Institute of Plant Protection, Xinjiang Academy of Agricultural Sciences, Urumqi 830000, China; fukaiyun000@foxmail.com; 3 Institute of Microbial Application, Xinjiang Academy of Agricultural Sciences, Urumqi 830000, China; gwc1966@163.com

**Keywords:** RNA interference, double-stranded RNA, sequence mutation, resistance development, Colorado potato beetle

## Abstract

RNA interference (RNAi)-based technology has been proven as a novel approach for insect pest control. However, whether insects could evolve resistance to RNAi and the underlying mechanism is largely unknown. The target gene mutations were thought to be one of the potential ways to develop the resistance. Here we predicted the effective siRNA candidates that could be derived from dsRNA against the Colorado potato beetle (CPB) *β-Actin* gene (ds*ACT*). By site-directed mutagenesis, we synthesized the dsRNAs with the defect in generation of effective siRNAs (and thus were supposed to have comparable low RNAi efficacy). We showed that, with mismatches to the target gene, all the dsRNA variants caused similar levels of silencing of target gene, mortality and larval growth retardation of CPB. Our results suggest that when the mismatch rate of ds*ACT* and target *β-Actin* mRNA is less than 3%, the RNAi efficiency is not impaired in CPB, which might imply the low possibility of RNAi resistance evolving through the sequence mismatches between dsRNA and the target gene.

## 1. Introduction

The Colorado potato beetle (CPB, *Leptinotarsa decemlineata* Say) is one of the most notorious pests in the world and may cause huge yield losses to potato production [1]. CPB has a complicated and diverse life history, a strong fertility, can adapt to adverse conditions, and has the capability to tolerate toxins [2,3], which make CPB control a big challenge [4]. Using chemical insecticides is still the major way to control CPB. However, high selection pressure and capability of tolerance to toxins had resulted in the outbreak of large number of insecticide-resistant CPB populations [5,6]. The insecticide resistance of CPB even promoted the development of the insecticide industry, with hundreds of chemicals against it [5]. Another alternative method is using *Bacillus thuringiensis* (Bt) toxins, which have potent and insecticidal activity. Pests can be killed by spraying insecticidal Bt toxins, or by expressing it in transgenic crops [7,8]. An increasing number of transgenic Bt crops have been grown globally for many years and have a very effective performance on pest control [9]. Transgenic potato plants expressing Bt Cry3Aa were highly effective in suppressing the population of CPB [10,11]. Yet, evolution of insect resistance to Bt crops may threaten the success of transgenic Bt crops. And pest resistance had emerged in fields where Bt crops were planted [12,13,14].

RNA interference (RNAi) is a post-transcriptional gene silencing mechanism induced by double-stranded RNA (dsRNA) and widely distributed in eukaryotes [15]. RNAi was employed as a useful tool to study gene functions [16], and also repurposed as a novel approach for pest control [17]. Administration of dsRNA targeted against the essential genes of pests could result in the silencing of target gene and influence the growth and fecundity of pests, and even lead to the death of pests. Numerous studies have indicated that transgenic plants expressing dsRNAs targeted against insect genes showed significant reduction in feeding damage [18,19]. By expressing dsRNAs targeted against the *β-Actin* gene of CPB in potato plastids, full protection was achieved as evidenced by all the CPB larvae dying after feeding on transplastomic potatoes [20]. In 2017, the US Environmental Protection Agency (EPA) approved the first plant-incorporated protectant based on RNAi technology (targeting the western corn rootworm, WCR, *Diabrotica virgifera virgifera* LeConte) which may approach the market soon [21,22]. It was known that genetic mutation in this population can be a major cause of insect resistance evolution for chemical insecticides and Bt toxins [5,23]. For example, mutations in genes encoding carboxylesterase or acetylcholinesterase could enhance insecticide resistance of insect pests [24]. A point mutation is responsible for the resistance to insecticides acting on ionotropic GABA (G-aminobutyric acid) receptors [25]. Mutations in ABC (ATP-binding cassette) transporters confer resistance to the Bt toxin Cry2Ab in several insects [26,27,28]. Resistance to the Bt toxin Cry1Ac was found to be conferred by mutations in the toxin-binding cadherin proteins [29,30]. Similar to insecticide resistance and Bt resistance, there is no doubt that insects would also evolve resistance to RNAi. Several mechanisms, including mutations of target genes or RNAi core machinery genes, may contribute to RNAi resistance [31]. Researchers established a WCR population resistant to maize expressing *DvSnf7* dsRNA and found that dsRNA uptake is changed in dsRNA-resistant WCR [32]. Another study found StaufenC, a dsRNA binding protein, is required for RNAi and is also a potential target for RNAi resistance [33]. These previous studies showed that the mutations of dsRNA uptake and processing could affect the RNAi responses of insects. 

In this study, we set out to explore whether sequence mutation of RNAi targeted genes could be one of the possible causes that result in the resistance to RNAi. By selectively mutating the most effective small interfering RNA (siRNA) sites, we synthesized six dsRNA variants to a 200 bp ds*ACT* and compared their insecticidal effects on CPB. We found that there was no significant reduction in RNAi efficacy induced by dsRNA variants compared with control.

## 2. Materials and Methods

### 2.1. Insect Material

CPB (*L. decemlineata*) larvae and adults were collected from a potato field in Urumqi (43.82 °N, 87.61 °E), Xinjiang Uygur Autonomous Region, China. CPBs were collected from the field and reared with wild-type potato plants in an insectary at 28 ± 1 ℃ and 50–60% relative humidity under a 14 h light–10 h dark photoperiod. Eggs from adult CPBs were collected and transferred onto fresh wild-type potato leaves before the bioassays.

### 2.2. In Vitro dsRNA Synthesis

The T7 RiboMAX^TM^ Express RNAi System (Promega, cat. No. P1700, Madison, WI, USA) was used for in vitro dsRNA synthesis according to the manufacture’s instruction. A cDNA sequence of the *ACT* gene was acquired from previous research [34]. A 200 bp DNA fragment, covering nucleotides +579 to +778 of the *ACT*-coding region, was PCR amplified using specific primers (Supplementary Table S1) containing the T7 promoter sequence. The other DNA fragments with mutations were synthesized (Genecreate, Wuhan, Hubei, China) and used as template for dsRNA synthesis with primers containing the T7 promoter sequence (Supplementary Table S1). The reaction mixture contained 2 μL T7 Express Enzyme Mix, 10 μL RiboMAX^TM^ Express T7 2 × Buffer, and 1 μg of DNA template. The mixture was incubated at 37 ℃ for 2–6 h and 70 ℃ for 10 min, then slowly cooled to room temperature (~20 min) to form the dsRNA. The DNA template and single-strand RNA (ssRNA) were removed by DNase (removing DNA template) and RNaseA (removing ssRNA) treatments, respectively. The yield of dsRNA was determined by a Nano Photometer (Implen, Munich, BY, Germany) and the integrity of the full length was detected by gel electrophoresis and stored at −80 ℃ until further use.

### 2.3. Insect Bioassays

The insect bioassays were performed as described previously [34]. Briefly, the third-instar CPB larvae were starved for 24 h before feeding assays. Equal amounts (16 ng, diluted in 50 μL ddH_2_O) of dsRNA were painted onto fresh potato leaves covering identical areas of 2 × 2 cm, thus the same dsRNA concentration of 4 ng cm^−2^ was obtained. ddH_2_O-painted leaves were used as control. Sufficient dsRNA-coated fresh leaves were supplied to insects and exchanged once per day. Three groups of insects per treatment were investigated, and each group contained 10 larvae. The body weight of larvae and the mortality were recorded daily and the *β-Actin* expression was detected at day 3.

### 2.4. Real-Time Quantitative PCR (qRT-PCR)

Total RNA samples were extracted using the RNAiso plus regent (Takara, Kyoto, Japan). Additional genomic DNA was digested by gDNA digester (Yeasen, Shanghai, China); 1.0 μg RNA was used for synthesizing cDNA by using the RevertAid First Strand cDNA Synthesis Kit (Yeasen, Shanghai, China). The *RP18* was chosen as the reference gene [35,36]. The 10 μL reaction mixture consisted of 5 μL TB Green Premix Ex Tap Ⅱ (TliRNaseH Plus) (Takara, Kyoto, Japan), 3.5 μL of nuclease-free water, 1 μL of cDNA, and 0.25 μL of forward and reverse primers (Supplementary Table S1). The qPCR protocol included an initial denaturation step at 95 ℃ for 2 min, followed by 40 cycles of 95 ℃ for 5 s, 60 ℃ for 30 s and 72 ℃ for 30 s. The relative expression of *β-Actin* was calculated by the 2^−ΔΔct^ method [37]. All experiments were repeated at least three times.

### 2.5. Statistical Analysis of Data

The effective candidates of siRNA were predicted by DNAMAN (v6.0.3.93) and two online tools: http://sidirect2.rnai.jp/ and https://www.invivogen.com/sirnawizard/design.php. The website for sequence alignment is http://multalin.toulouse.inra.fr/multalin/. All parameters are default parameters, such as: (i) A/U (adenine/uracil)at the 5’ end of the antisense strand; (ii) G/C (guanine/cytosine) at the 5’end of the sense strand; (iii) at least five A/U residues in the 5’ terminal one-third of the antisense strand; and (iv) the absence of any GC stretch of more than 9 nt in length [38]. The Kaplan-Meier method was used in the survival curves analysis. The log-rank test was used to evaluate the significance of differences between the two groups. Data of qRT-PCR and body weight were analyzed with one-way ANOVA coupled with Bonferroni’s (equal variances) or Dunnett’s T3 (unequal variances) correction multiple comparison test. A value of *p* < 0.05 was considered significantly different. Data were statistically analyzed by SPSS version 19.0. Figures were drawn by GraphPad Prism 7 and SnapGene 3.2.1.

## 3. Results

### 3.1. Introduction of Point Mutations with Potentially Impaired Generation of Effective siRNAs on RNAi Efficacy in Colorado Potato Beetle

**Figure 1:**
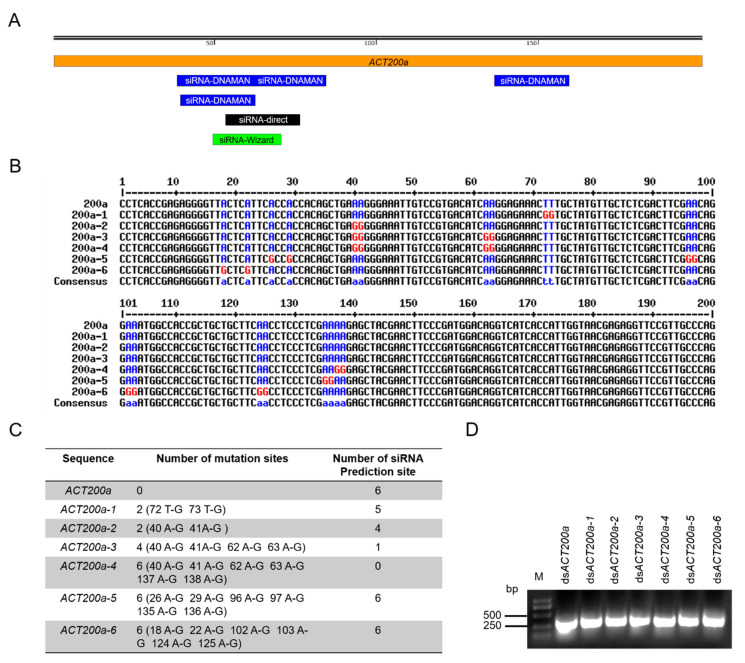
Introduction and in vitro synthesis of ds*ACT*s with point mutations. (**A**) The relative location of effective siRNA candidates at *ACT200a* which were predicted by indicated bioinformatic toolboxes (DNAMAN, siDirect and siRNA Wizard). (**B**) Alignment of original *ACT200a* sequence with mutated *ACT200a* variants. The high consensus base pairs are represented as blue. The mutated base pairs are indicated as red. (**C**) Summary of the number and location of mutations in (**B**). (**D**) Analysis of the synthesized ds*ACT*s by electrophoresis. M, 1 kb DNA ladder.

A total of six siRNA sites were predicted to be generated from a 200 bp of *ACT* fragment (*ACT200a*) (Figure 1A). Given that the mutation of the target gene sequence would result in the RNAi resistance of insects, there was a high possibility that mutations would occur at the sites which have the potential to derive those effective siRNAs. Therefore, we introduced the point mutations into regions of *ACT200a* that impaired the effective siRNA generation by T-G or A-G mutation (Figure 1B). For comparison, point mutations outside the effective-siRNA-generation region were introduced as control. Six ds*ACT200a* variants were synthesized. ds*ACT200a-1* and ds*ACT200a-2* had mutations to generate five and four effective siRNAs, respectively. Mutations introduced in ds*ACT200a-3* resulted in the generation of only one effective siRNA, whilst ds*ACT200a-4* could not generate effective siRNAs. In ds*ACT200a*-5 and ds*ACT200a*-6, mutations were introduced to the sites which did not interfere the generation of effective siRNAs and served as control (Figure 1C). Six ds*ACT200a* variants plus the original ds*ACT200a* were in vitro synthesized and detected by gel electrophoresis. (Figure 1D and Figure S1).

### 3.2. Effect of dsACT with Different Mutations on RNAi Efficacy in Colorado Potato Beetle Larvae

**Figure 2:**
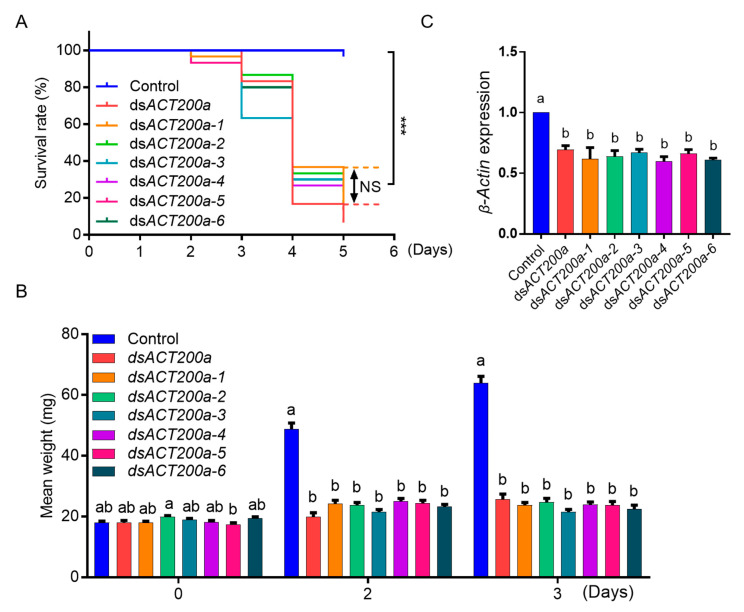
RNAi effects on Colorado potato beetle (CPB) larvae fed with different in vitro synthesized dsRNA variants. (**A**) Kaplan-Meier survival curves of third-instar CPB larvae fed with potato leaves that had been painted with identical amounts (4 ng/cm^2^) of indicated ds*ACT*s. The log-rank test was used to assess the significance of differences between two survival curves. *** *p* < 0.001; NS, not significant. (**B**) Mean weight of surviving CPB larvae at the indicated days of feeding. Data are means ± SE (n = 30). (**C**) Relative expression levels of *β-Actin* in the CPB larvae in (**A**) at day 3. Gene expression levels were set as one in CPB larvae fed with H_2_O-painted control leaves. Data are means ± SE (n = 3). The letters above each bar in (**B**,**C**) indicate the significance of differences as determined by one-way ANOVA in SPSS (Bonferroni’s test, *p* < 0.05).

**Figure 3:**
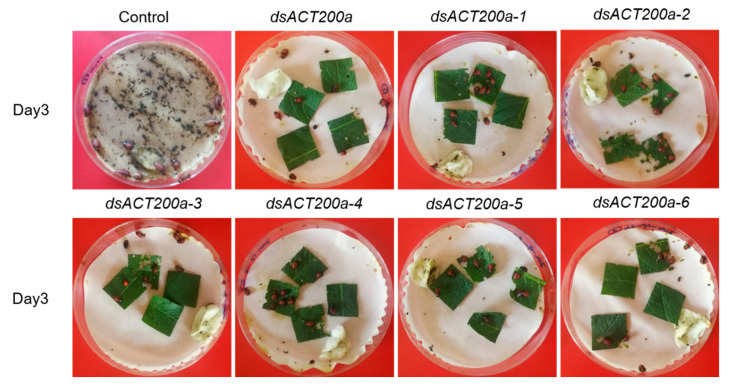
Leaves painted by H_2_O and ds*ACT200a* and its mutations were fed to third-instar CPB after 3 days.

We next tested whether the mutations of ds*ACT* could affect the RNAi efficacy in CPB. Potato leaves painted with d*sACT* were fed to third-instar larvae of CPB. Compared with the control (fed with H_2_O-painted leaves), CPB larvae fed with all the ds*ACT*-treated leaves had increased mortality, inhibited growth and suppressed *β-Actin* expression (Figure 2A–C). However, no significant differences in mortality, weight gain of survivors and level of gene downregulation were observed among the seven ds*ACTs* treatments (Figure 3). These results revealed that the mutations introduced into ds*ACT* sequences did not markedly affect RNAi efficiency in controlling CPB.

## 4. Discussion

RNAi technology has been proven as an effective strategy for CPB control [20,39]. Whether and how CPB develops resistance to RNAi remains elusive. In the RNAi pathway, dsRNAs can be processed by Dicer endoribonuclease into 21–23 bp siRNAs, which then are loaded into the RNA-induced silencing complex (RISC) to cleave the target mRNA in a sequence-specific manner [40]. It is critical that the guide strand of siRNA is complementary to the target mRNA, while the mismatch of siRNA to the target mRNA might result in off-target effects and thus impede the RNAi efficiency. Under high pressure of insecticidal dsRNA, it is possible that CPB would accumulate the mutations at the target gene that were targeted by this specific dsRNA to reduce the RNAi efficiency. Sequence mutation is one direction of evolving resistance for CPB to RNAi.

Previously, we showed that transplastomic potato expressing dsRNA against *β-Actin* genes acquired high resistance to CPB [20]. Recently, naturally occurring mutations of western corn rootworm with resistance to RNAi was first reported in a transgenic corn field screen with subsequent laboratory selection. It showed that DvSnf7-dsRNA resistant WCR was also cross resistant to other dsRNAs, and dsRNA uptake rather than degradation was responsible for evolution of RNAi resistance [32]. This prompted us to investigate whether RNAi resistance could also occur in CPB fed on the *dsACT*-expressing transplastomic potato plants. Gene mutations are one of the driving forces to evolve the resistance. Potentially, target *β-Actin* gene mutation could be the resource of RNAi resistance. Under this circumstance, sequence mismatches between dsRNA and target genes could lead to the off-target effects. Since the gene editing tools for introducing mutations to the target gene are not available, we instead introduced the mutations to dsRNAs to mimic the mismatches between the dsRNA and target gene. Our results showed that ds*ACT*s with sequence mutations were still effective and had no significant difference compared to the original ds*ACT*, which demonstrated that the mismatches of dsRNA to target mRNA could not affect the RNAi efficiency in CPB. It was found that the target recognition process is highly sequence specific in the RNAi pathway, but not all positions of siRNA contribute equally to target recognition in a *Drosophila melanogaster* embryo lysate [41]. The sequence mutation rates of ds*ACT200* variants were all under 3%, so whether a higher mutation rate (>3%) could affect the RNAi efficiency remains to be investigated. It also should be mentioned that mutations introduced to ds*ACTs* were based on siRNA prediction programs, which could not identify all the effective siRNAs with algorithms. Therefore, the position of a mutation may not be the key nucleotide for the generation of all the effective siRNAs.

## 5. Conclusions

Taking this together, our study demonstrates that the mismatch rate of dsRNA and a target mRNA lower than 3% in CPB does not reduce the RNAi efficiency in CPB, which may imply that the possibility of RNAi resistance evolving by sequence mutation of target genes would be low. This study will lay the foundation for studying the evolution of RNAi resistance in CPB.

## Supplementary Materials

The following are available online at https://www.mdpi.com/2075-4450/11/7/449/s1, Figure S1: The relative location of potential sites were predicted to generate effective siRNAs after mutation introduction to ACT*200a* (ACT*200a-1* to ACT*200a-6*), Table S1: List of oligonucleotides used in this study.
