# Supplementary material for: Low Mismatch Rate between Double-Stranded RNA and Target mRNA Does Not Affect RNA Interference Efficiency in Colorado Potato Beetle
Source: Insects. 2020 Jul 16;11(7):449. doi: 10.3390/insects11070449 (PMC7411949; doi:10.3390/insects11070449)
Supplement: Supplementary file 1 [file insects-11-00449-s001.pdf]

insects MDPI

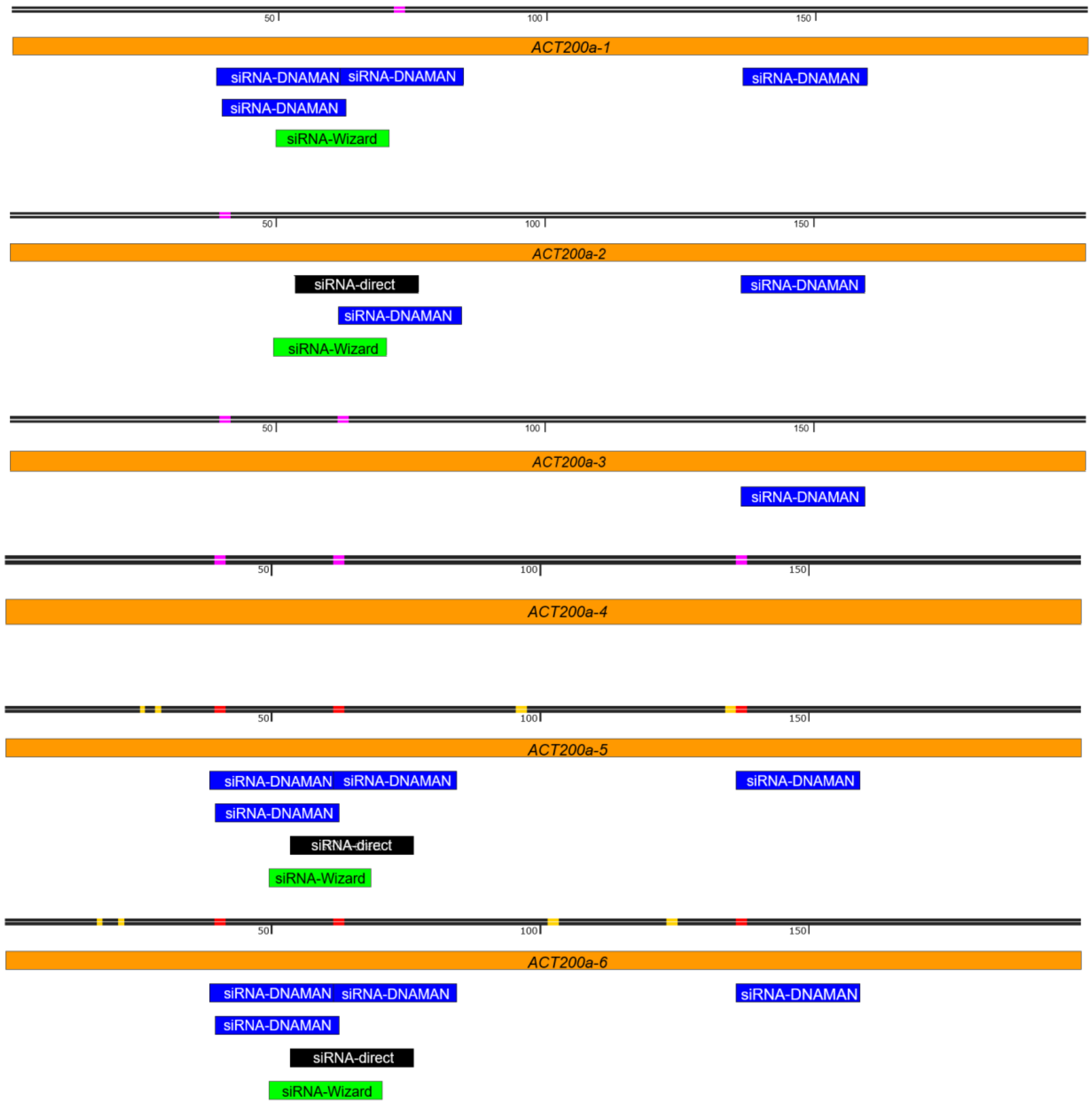


**Figure S1.** The relative location of potential sites were predicted to generate effective siRNAs after mutation introduction to *ACT200a* (*ACT200a-1* to *ACT200a-6*).

**Table S1.** List of oligonucleotides used in this study.

| Oligonucleotide | Sequence (5′-3′) | Description and Use |
|---|---|---|
| T7act200a-Fwd | GGATCCTAATACGACTCACTATAGGCCTCACCGAGAGGGGTTACTC | forward primer for amplification of the *ACT200a* fragment; introducing the T7 promoter sequence; for in vitro dsRNA synthesis |
| T7act200a-Rev | GGATCCTAATACGACTCACTATAGGCTGGGCAACGGAACCTCTCG | reverse primer for amplification of the *ACT200a* fragment; introducing the T7 promoter sequence; for in vitro dsRNA synthesis |
| T7act200-MFwd | GGATCCTAATACGACTCACTATAGGCCTCACCGAGAGGGGTT | forward primer for amplification of the *ACT200a-1* to *ACT200a-6* fragments; introducing the T7 promoter sequence; for in vitro dsRNA synthesis |
| T7act200-MRev | GGATCCTAATACGACTCACTATAGGCTGGGCAACGGAACCTCTC | reverse primer for amplification of the *ACT200a-1* to *ACT200a-6* fragments; introducing the T7 promoter sequence; for in vitro dsRNA synthesis |
| qRT-ACT-F | TGCAGAAGGAAATCACCGCT | forward primers for qRT-PCR analysis of *ACT* expression |
| qRT-ACT-R | CACTTGCGGTGAACGATTCC | reverse primers for qRT-PCR analysis of *ACT* expression |
| qRT-RP18-F | TAGAATCCTCAAAGCAGGTGGCGA | forward primers for qRT-PCR analysis of *RP18* expression (as reference gene) |
| qRT-RP18-R | AGCTGGACCAAAGTGTTTCACTGC | reverse primers for qRT-PCR analysis of *RP18* expression (as reference gene) |
